# *In situ* Dynamics of O_2_, pH, Light, and Photosynthesis in Ikaite Tufa Columns (Ikka Fjord, Greenland)—A Unique Microbial Habitat

**DOI:** 10.3389/fmicb.2016.00722

**Published:** 2016-05-19

**Authors:** Erik C. L. Trampe, Jens E. N. Larsen, Mikkel A. Glaring, Peter Stougaard, Michael Kühl

**Affiliations:** ^1^Marine Biological Section, Department of Biology, University of CopenhagenHelsingør, Denmark; ^2^Section for Microbial Ecology and Biotechnology, Department of Plant and Environmental Sciences, University of CopenhagenFrederiksberg, Denmark; ^3^Plant Functional Biology and Climate Change Cluster, Department of Environmental Sciences, University of Technology SydneySydney, NSW, Australia

**Keywords:** ikaite, tufa columns, photosynthesis, microsensor, biofilm, extreme environments, alkaline

## Abstract

The Ikka Fjord (SW Greenland) harbors a unique microbial habitat in the form of several hundred submarine tufa columns composed of ikaite, a special hexahydrate form of calcium carbonate that precipitates when alkaline phosphate- and carbonate-enriched spring water seeping out of the sea floor meets cold seawater. While several unique heterotrophic microbes have been isolated from the tufa columns, the microbial activity, and the boundary conditions for microbial growth in ikaite have remained unexplored. We present the first detailed *in situ* characterization of the physico-chemical microenvironment and activity of oxygenic phototrophs thriving within the ikaite columns. *In situ* underwater microsensor measurements of pH, temperature, and irradiance in the porous ikaite crystal matrix, revealed an extreme microenvironment characterized by low temperatures, strong light attenuation, and gradients of pH changing from pH 9 at the outer column surface to above pH 10 over the first 1–2 cm of the ikaite. This outer layer of the freshly deposited ikaite matrix contained densely pigmented yellow and green zones harboring a diverse phototrophic community dominated by diatoms and cyanobacteria, respectively, as shown by amplicon sequencing. *In situ* O_2_ measurements, as well as underwater variable chlorophyll fluorescence measurements of photosynthetic activity, demonstrated high levels of oxygenic photosynthesis in this extreme gradient environment with strong irradiance-driven O_2_ dynamics ranging from anoxia to hyperoxic conditions in the ikaite matrix, albeit the local formation of gas bubbles buffered the day-night dynamics of O_2_ in the tufa columns. The microbial phototrophs in the ikaite matrix are embedded in exopolymers forming endolithic biofilms that may interact with mineral formation and cementing of ikaite crystals.

## Introduction

The Ikka Fjord in southwestern Greenland harbors a unique assemblage of tufa columns found nowhere else on the planet (Buchardt et al., 1997; Seaman and Buchardt, 2006). Columns formed by the special carbonate mineral ikaite protrude from the seabed in sizes varying from a few centimeters to more than 20 m in height and several meters wide (Pauly, 1963). The largest columns are visible from the shore and are just breaking the surface during extreme low spring tides. Using detailed side-scan sonar mapping, Seaman and Buchardt (2006) found 657 columns of >1 m height in Ikka Fjord, and they dated the oldest columns to have formed ~10,000 years ago. While scientific exploration of these columns is much more recent, the first mention of their existence occurs in old Inuit legends, where the ikaite tufas are regarded as remnants of Norsemen standing on the fjord bottom after falling through the ice during a chase by the Inuit people (Rink, 1866). The occurrence of ikaite in nature was first described and named after the fjord in 1963 (Pauly, 1963). Further investigation of the columns was not initiated until 1995 and has since continued intermittently through a number of summer and winter fieldtrips encompassing geological, zoological, botanical, and microbiological studies.

The tufa columns are mainly composed of the mineral ikaite, which is a hexa-hydrated calcium carbonate (CaCO_3_·6H_2_O). The mineral has also been found as minor deposits in other cold habitats e.g., in sea-ice, precipitating during the freezing process of seawater (Dieckmann et al., 2008, 2010), and as a minor part in a seasonal tufa mound formation by the shores of Mono Lake in California (Whiticar and Suess, 1998). Recently, massive ikaite formation was also observed in artificial alpine riverbeds (Boch et al., 2015). However, in the mentioned habitats ikaite mainly forms a crystal ooze, and the solid submarine column formation is unique and only found in the Ikka Fjord, which has been suggested to be classified as a geological World Heritage Site (Buchardt et al., 1997; Seaman and Buchardt, 2006).

In Ikka Fjord, meteoric melt- and rain-water seeps through the surrounding ~500 m steep, Precambrian porous carbonatite rock formations (Buchardt et al., 1997). During this seepage, the water interacts with the rock minerals and becomes highly alkaline (pH 10–11) and enriched in carbonate, bicarbonate and phosphate (Buchardt et al., 1997). The alkaline water is forced out below the seabed, and released through submarine seeps in the inner Ikka Fjord bottom, where it is mixed with cold (< 6°C) seawater triggering ikaite crystallization. Slow recrystallization of ikaite to calcite at the column periphery and partial sealing of the porous ikaite matrix by coralline algae create hydraulic forcing and a continuous flow of alkaline spring water from the seabed, creating chimney-like flow channels within the ikaite matrix (Hansen et al., 2011). The density of the different water masses dictates column formation and direction of growth. The lighter spring water (salinity ~9 psu; Buchardt et al., 1997) is forced upwards by the heavier seawater with a salinity of ~31 psu. This favors rapid column growth at the apex of columns, where porous ikaite is deposited year-round at rates of up to 4–5 cm per month (Hansen et al., 2011). The process of column formation is mostly considered to be driven by the abiotic chemical crystallization process, albeit the exact mechanisms governing the formation of the highly structured ikaite matrix in the columns remain unknown (Pauly, 1963; Buchardt et al., 1997; Seaman and Buchardt, 2006).

The ikaite columns are colonized by various epilithic organisms such as bivalves, snails, tunicates, sea anemones, sea urchins as well as tubeworms and encrusting algae, which together with coralline algae provide structural stability to the surface of the otherwise brittle ikaite (Buchardt et al., 1997; Kristiansen and Kristiansen, 1999; Sørensen and Kristensen, 2000). Thorbjørn and Petersen (2003) recorded 116 macrofauna species associated with ikaite columns, and the column gardens may also serve as a protected habitat or nursery ground for juvenile fish as found for other marine reef structures around the world.

Most microbiological studies of the ikaite columns have focused on the bacterial diversity with special emphasis on isolating heterotrophic bacteria that might harbor cold-active enzymes of industrial interest (Schmidt et al., 2006; Schmidt and Stougaard, 2010; Schmidt et al., 2010; Vester et al., 2014). Novel alkaliphilic and psychrophilic heterotrophic bacteria have been isolated from the ikaite columns, several of which have resulted in findings of novel enzymes of biotechnological importance (Schmidt et al., 2007; Vester et al., 2014). Molecular phylogenetic analyses on older and/or deeper parts of ikaite columns have revealed a wealth of mostly uncultivated eukaryotes and prokaryotes (Stougaard et al., 2002; Vester et al., 2013; Glaring et al., 2015) but so far no *in situ* measurements of microbial activity have been attempted, and speculations about the metabolic potential of microbes in the ikaite columns have solely been based on phylogenetic comparisons to other microbes with known metabolic capabilities.

Blue-green pigmented patches inside the columns were first described by Kristiansen and Kristiansen (1999), along with the presence of the microalga *Chroomonas ikaitensis*. Yellow-brown patches inside the columns were later identified to originate from the diatom, *Surirella brebissonii* (Sørensen and Kristensen, 2000). The presence of such patches may reflect adaptations to particular microhabitats driven by chemical zonations in the ikaite matrix and/or light microhabitats. However, proof of active photosynthesis and a more detailed analysis of microbial phototrophs in the ikaite columns has not been provided, and their physical and chemical microenvironment in the ikaite matrix remains largely unexplored (Hansen et al., 2011).

In this study, we present the first detailed *in situ* investigation of the physico-chemical microenvironment and oxygenic photosynthesis of microbial phototrophs colonizing the freshly-deposited ikaite at the light-exposed apex of ikaite tufa columns. We combine the studies with molecular analyses of the phototrophic community present in this extreme and unique microbial habitat.

## Methods

### Study site

**Figure 1:**
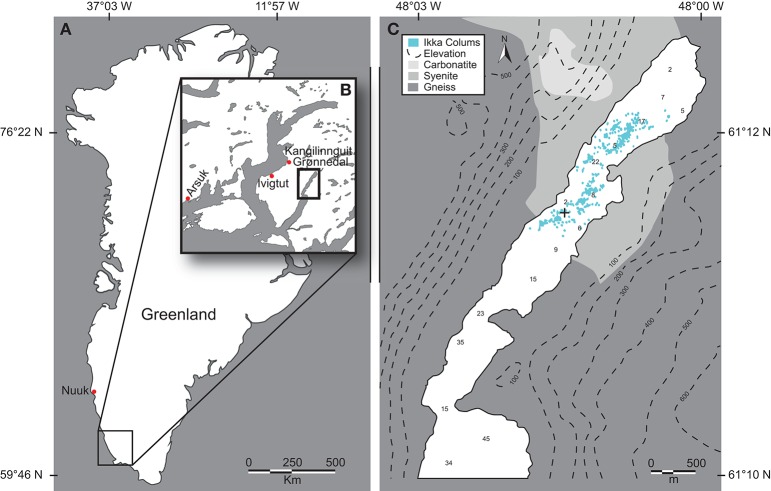
**Ikka Fjord**. **(A)** Map of Greenland with Ikka Fjord located in the SW. **(B)** Ikka Fjord and neighboring fjords. **(C)** Insert from **(B)**, showing ikaite column distribution, and a coarse overview of the mineral contents of the surrounding mountain complexes, with topography displayed by isobars and distribution of ikaite tufas by blue dots (after Seaman and Buchardt, 2006). “+” denotes the location of the investigated tufa column.

**Figure 2:**
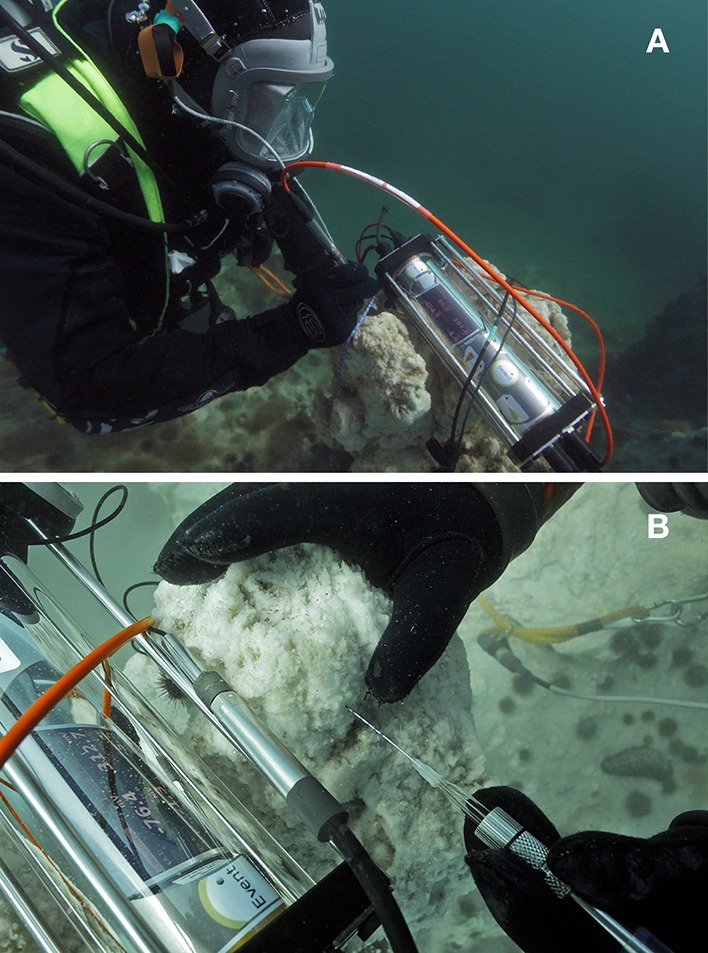
**Ikaite columns. (A)** Diver performing underwater microprofile pH measurements in the upper photic part of the column with its apex situated at a depth of 7 m below mean sea level. **(B)** Close-up photo showing diver-operated measurements with a needle O_2_ sensor connected to an underwater microsensor meter. The sensor was carefully inserted into the porous ikaite matrix by the diver using markings on the needle sensor as indicators of measuring depth.

Data and samples were collected over several summer field-campaigns 2008–2013 due to the remote location and complicated logistics for diver-based work; most *in situ* studies were conducted in August 2012 and 2013 on the same specific tufa column in Ikka Fjord, SW Greenland (61°11.528′N 48°01′662′W). The Ikka Fjord is long and narrow with a maximum depth of 30 m oriented from NE-SW in a glacial valley bordered by steep, ~500 m high Precambrian mountains mainly composed of carbonatite, syenite, and gneiss. Tufa columns are only found in the shallow inner part of the fjord, “Ikka Bund,” that is protected by a geological land barrier forming a narrow entrance to the inner fjord (Figure 1); this barrier renders column formation unaffected by icebergs entering the outer fjord. A tidal range of ~3 m provides a large exchange of water, where seawater enters the fjord and partly mixes with the freshwater from the submarine springs and the many surrounding terrestrial streams leading into the inner fjord. All underwater measurements were performed by certified commercial scuba divers investigating a single ikaite column during ~25 dives with additional dives for sampling and documentation. The investigated column was branching off from a bigger column that belongs to a range of columns on a ridge protruding from one of the most massive ikaite formations “The Atoll,” oriented toward NNE (Figures 1, 2).

**Figure 3:**
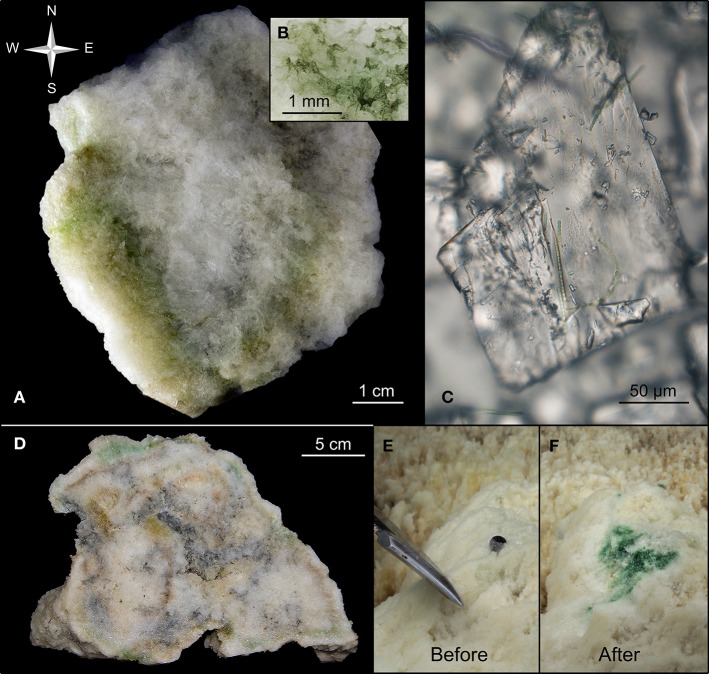
**Biofilm layers in ikaite. (A)** Cross-section of an ikaite column with dense green and yellowish bands of various thickness along the surface, **(B)** close-up of biofilm, **(C)** close-up of ikaite crystal with filamentous cyanobacteria, **(D)** cross-section of several ikaite columns fused together, with dense green and yellowish bands along the surface, **(E,F)** *In situ* exposure of green biofilm in ikaite. **(E)** Intact ikaite surface with the tip of a knifeblade. **(F)** Sub-surface pigmented layer and small gas bubbles revealed after the outermost ~15 mm thick ikaite layer was scraped of.

The upper photic part of the column had its apex situated at a depth of ~7 m at mean sea level. The column apex exhibited persistent and rapid formation of fresh ikaite crystals. A continuous flow of meteoric water seeping from its porous ikaite matrix created visible clouding from spring water mixing with the surrounding seawater, and rapid re-crystallization in cuts from harvested samples was observed. A small flat platform shaped the column apex, measuring ~45 × 25 cm, with three minor, 10–15 cm wide and ~5 cm high protruding freshly deposited ikaite formations (Figure 2). After completion of the *in-situ* measurements, the top of the investigated column was cut off with a saw and cross sections showed the presence of distinct green and yellowish colored zones of variable thickness in the ikaite matrix (Figure 3).

### Light microscopy

Images of the ikaite crystal matrix and associated microbes (Figures 3B,C) were obtained from the freshly harvested ikaite column top by a digital SLR camera (EOS 7D digital SLR camera, CANON Europe Ltd., Middlesex, UK) fitted on the camera port of an epifluorescence microscope with 5x and 20x objectives (Axiostar Plus FL microscope with Plan-Apochromat objectives, Carl Zeiss GmbH, Germany).

### *In situ* light and temperature measurements

Light measurements of photon scalar irradiance (PAR; 400–700 nm) at the ikaite surface were performed *in-situ* with a spherical micro quantum sensor (US-SQS/A, Heinz Walz GmbH, Effeltrich, Germany) connected to an underwater microsensor meter (Underwater Meter System, Unisense A/S, Århus, Denmark). Light measurements were done in parallel with, and in close proximity to, the site of O_2_ and pH profile measurements (see below). Photon irradiance (PAR) at the water surface was monitored by a universal light meter (ULM-500, Heinz Walz GmbH, Effeltrich, Germany).

Temperature loggers (iButton®–DS1922L, Maxim Integrated, San Jose, CA, USA) were placed on the ikaite column at three different depths in August 2013 recording temperature at an interval of 5 min for 7 days. The temperature loggers were placed at the top platform of the column of interest, at ~7 m mean sea level, and 1.5 m above and below this depth, Tidal data of the neighboring fjord Kangilinnguit (Grønnedal), were obtained from the Danish Meteorological Institute (www.dmi.dk) for the same period as temperature recording was performed.

### *In situ* pH and O_2_ measurements

The chemical conditions in the ikaite matrix were measured by detailed mapping of O_2_ concentrations and pH in the outermost layers of the column apex. Profiles of O_2_ and pH were obtained *in situ* by the use of underwater pressure-compensated Clark-type O_2_ needle electrodes (OX-N, Unisense A/S; Revsbech, 1989) and pressure-compensated pH glass needle electrodes with a separate silver/silver-chloride open-ended reference macroelectrode (pH-N, Unisense A/S). The electrodes were connected to a four-channel underwater microsensor meter (Underwater Meter System, Unisense A/S, Århus, Denmark). The O_2_ electrodes were linearly calibrated at *in situ* water temperature from signal readings in aerated seawater and in seawater made anoxic by addition of sodium dithionite. The pH electrodes were calibrated at *in situ* water temperature by a two-point calibration in standard pH buffers (pH 7 and 10).

The needle sensor measurements were carried out handheld by a diver, who could carefully insert the needle sensors into the porous ikaite matrix in steps of 5 mm, following markings on the needle tips (Figure 3). Profiling was done from the column surface to a depth of ~15–25 mm inside the column. Due to the fragility of the pH-electrodes, most pH measurements only reached a maximum profile depth of 15 mm, as the sensor glass tip was frequently damaged when attempting to go deeper. No repetitive measurements were done in the same sensor “hole,” and all individual measurements were thus done in separate positions on the apex of the ikaite column.

Concentration profiles of O_2_ were measured at varying orientations on the tufa column relative to the incident sun light, to map any differences in O_2_ in relation to changes in the prevailing incident light conditions. The measured concentration profiles thus represent O_2_ levels at different light environments from the surface of the column to a depth of 20–30 mm within the matrix. All measuring points were sorted according to their orientation on the column, yielding profiles at different sun angles relative to the column apex, i.e., east, south, west, north, and exposed (top). The definition of N, S, E, and W was done by the divers compass. Measurements within an orientation zone were obtained in a line going down the column with a few cm between measurements on the side of the column, whereas top measurements were obtained more randomly on the top of the column. For each profile measurement, the microsensor was inserted normal to the ikaite surface. The measured O_2_ profiles were further separated according to time of day; when the sun was at its highest around noon, and in darkness 1–3 h after sunset, and between 2 years (August 2012 and 2013).

Diel *in-situ* dynamics of O_2_ concentration and photon scalar irradiance were measured during two 24 h cycles; one diel cycle with 100% cloud cover (completely overcast), and one diel cycle with 0% cloud cover (blue sky). During these measurements, the tip of the O_2_ needle sensor was fixed at a depth of ~15 mm inside the ikaite matrix, yielding a relatively high O_2_ reading at the beginning of a cycle. The photon scalar irradiance sensor was positioned on the ikaite surface in close proximity to the insertion point of the O_2_ electrode. About 4–5% of the incident irradiance remained at a depth of 15 mm, as calculated from the ikaite surface light measurements obtained in this study and light attenuation coefficients of ikaite measured in the laboratory (Trampe et al., unpublished data).

### Variable chlorophyll fluorescence

The effective quantum yield of photochemical energy conversion via photosystem (PS)II activity in the ikaite matrix was assessed *in-situ* under natural light conditions by a submersible, diver-operated pulse-amplitude modulated (PAM) fluorometer (Diving-PAM, Heinz Walz GmbH, Effeltrich, Germany; Schreiber et al., 1997; Kühl et al., 2001) in August 2013. The fluorometer was equipped with a fiber-optic cable (Ø 5.5 mm; DIVING-F, Heinz Walz GmbH, Effeltrich, Germany) that was directed toward the ikaite crystal matrix harboring surface-associated phototrophs for monitoring chlorophyll fluorescence yields with a weak non-actinic measuring light. From measurements of the fluorescence yield before and during a strong saturating light pulse, the effective quantum yield of PSII (ϕ_*PSII*_), equivalent to the fraction of energy that is photochemically converted in PSII, was determined as (Genty et al., 1989):

$$\begin{array}{l} \phi_{PSII}=(F_{m}\prime -F\prime )/F_{m}\prime \end{array}$$

where *F*′ is the fluorescence yield measured under ambient light conditions, and *F*${}_{m}^{\prime }$ is the maximal fluorescence yield measured during the saturation pulse. Subsequently, the relative photosynthetic electron transport rate (rETR) was calculated as:

$$\begin{array}{l} rETR=\phi_{PSII}\;\times \;E\left(PAR\right)\times 0.5 \end{array}$$

where *E(PAR)* is the incident photon irradiance of ambient photosynthetically active radiation (400–700 nm) and the constant 0.5 is from the assumption that the photon energy absorbed is equally divided between *PSII* and *PSI*. The *ETR* rates are relative since we did not measure the absorption cross-section of PSII.

It was possible to automate measurements of rETR under a series of increasing levels of actinic irradiance as provided via the fiber-optic light cable. With a short (10 s) exposure to each irradiance level before a measurement pulse, so-called rapid light curves (RLC) of PSII activity vs. photon irradiance (Ralph and Gademann, 2005) were recorded, representing a snapshot of the current light acclimation status of the monitored photosynthetic community in the ikaite matrix. In contrast, measuring pulses recorded with longer incubation periods (>5–10 min) at each irradiance yield steady state light curves (SLC), where the photosynthetic apparatus is allowed to acclimate to increasing irradiance. In this study, we focused on RLC measurements to document the *in situ* light acclimation of the phototrophs in the ikaite matrix. The light saturation coefficient, i. e., the irradiance at onset of light saturation of photosynthesis was calculated as *E*_*k*_ = *rETR*_*max*_∕α, where *rETR*_*max*_ is the maximum activity and α is the initial slope of the rETR vs. irradiance curve; both parameters were obtained by curve fitting of rETR vs. irradiance curves with an exponential function (Webb et al., 1974) using a non-linear Levenberg-Marquardt fitting algorithm in OriginPro 2015 (OriginLab Corporation, Northampton, MA, USA).

Before *in situ* measurements of variable chlorophyll fluorescence, dense green patches inside the ikaite column apex were revealed by carefully scraping off the outermost ~2–6 mm of white ikaite with a sharp knife (Figures 3E,F). The fiber-optic cable from the Diving-PAM was then placed by the diver a few mm above the green patch (Supplementary Figure 1), and ϕ_*PSII*_ in the exposed pigmented ikaite matrix was measured as a function of nine consecutive increasing photon irradiances of actinic light, following a custom pre-programmed and calibrated (PAR) list of photon irradiance levels ranging from 0 to 258 μmol photons m^−2^ s^−1^.

### Pyrosequencing of 16S rRNA genes

Ikaite samples for molecular analysis of the microbial diversity were retrieved by collecting ikaite material from distinct colored zones in a larger cross section taken from the column apex. The microbial diversity in the samples was analyzed by pyrosequencing of 16S rRNA genes, and sequence analysis was carried out as previously described (Glaring et al., 2015). Briefly, DNA was extracted from ikaite material using a MO-BIO Powersoil DNA Extraction Kit (MO-BIO Laboratories, Carlsbad, CA, USA). A 466 bp fragment of the 16S rRNA gene from bacteria and archaea covering the V3 and V4 hypervariable regions was PCR amplified using the primers 341F (5′-CCTAYGGGRBGCASCAG-3′) and 806R (5′-GGACTACNNGGGTATCTAAT-3′). Adapters and tags for pyrosequencing were added to the gel-purified PCR products in a second PCR using the same primers carrying sequencing adapters and tags for multiplexing. The amplified fragments were sequenced on a Genome Sequencer FLX pyrosequencing system (454 Life Sciences, Roche, Branford, CT, USA).

Trimming and quality-filtering of 16S rRNA gene sequences was performed using the software suite Biopieces (www.biopieces.org). OTUs were clustered at 97% sequence identity using USEARCH (Edgar, 2010) and chimeric OTUs were removed using the UCHIME function by comparison to the chimera-free RDP Gold database (http://drive5.com/uchime/rdp_gold.fa). OTUs containing only one sequence (singletons) were discarded. Phylogenetic analysis was performed using the Quantitative Insights into Microbial Ecology (QIIME) pipeline version 1.9.1 (www.qiime.org) (Caporaso et al., 2010) by comparison to the Greengenes database (version 13_08; http://greengenes.secondgenome.com). In this study, we mainly focused on analyzing the microbial phototrophs (cyanobacteria and microalgal plastids) in ikaite. The complete dataset of 92,266 quality-filtered sequences in QIIME-compatible format is available from the MG-RAST server (http://metagenomics.anl.gov; ID 4653755.3).

## Results

### Temperature and tidal conditions

**Figure 4:**
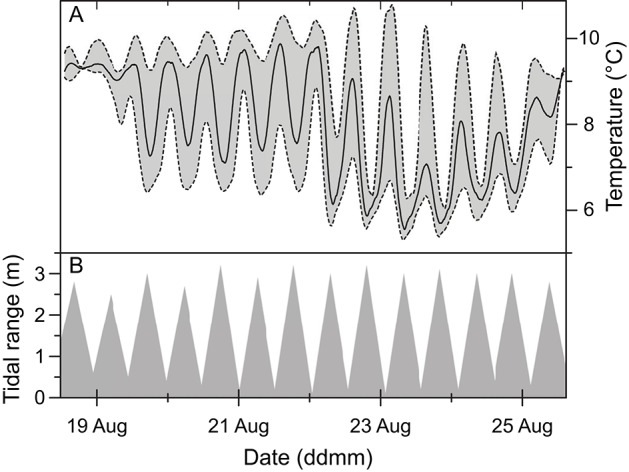
**Temperature data (A) plotted on a 7-day time scale from dataloggers placed on the apex of an ikaite tufa column at a depth of 7 m at mean sea level, and 1.5 m above (upper-dashed line) and below (lower dashed line) the column apex, presented along with tidal data (B)**.

Temperature measurements recorded at three different depths in 2013, showed a dynamic temperature variation at the tufa column surface that correlated with tidal changes (Figure 4A). Under rising water level at incoming tide, temperature decreased due to cold oceanic seawater entering and mixing with the warmer fjord water. This effect was reversed during outgoing tides, where relatively warmer surface waters reached the measuring location. During the 7 days of measurements, the recorded temperature mean at the column apex was 7.5°C with a maximum span of 4.4°C and maximum and minimum temperatures of 9.4 and 5.0°C, respectively. The average temperature variation during a tidal cycle was 2°C, with a maximum and minimum variation of 3.6 and 0.2°C, respectively. The tidal data did not take other factors such as changing wind and currents into account (Figure 4B).

### pH microenvironment

**Figure 5:**
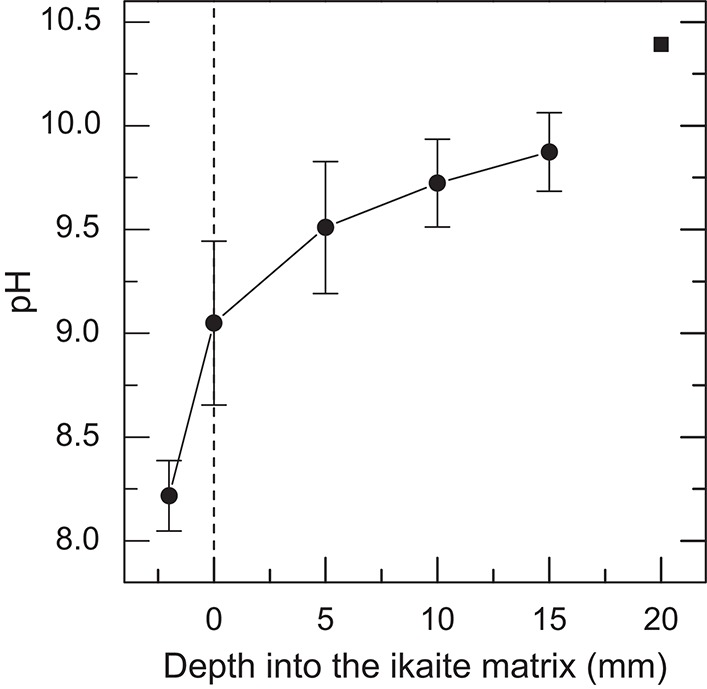
***In situ* profiles of pH measured along a depth gradient into the ikaite matrix**. Data were obtained during the field campaign in 2012. Symbols with error bars indicate Means ± SE (*n* = 32); the pH value at 20 mm is represented by a single measurement.

All 32 pH profile measurements displayed similar characteristics despite being obtained from various locations on the column relative to sun angle and orientation (facing south, north, east, west and up) at various times of day, and thus shifting irradiance levels (data not shown). Thus, all pH measurements were pooled and plotted as the mean pH (Figure 5). The pH profiles revealed a strong increase from pH 8.25 in the seawater 2 mm above the ikaite surface to pH 9.1 at the porous ikaite surface, followed by a more gradual increase in pH vs. depth reaching pH 9.9 about 15 mm inside the ikaite matrix. It was very difficult to go deeper inside the ikaite crystal matrix without fracturing the delicate tip of the pH sensor, and the shown data point reaching pH 10.4 at a depth of 20 mm thus represents a single measurement.

### O_2_ microenvironment

**Figure 6:**
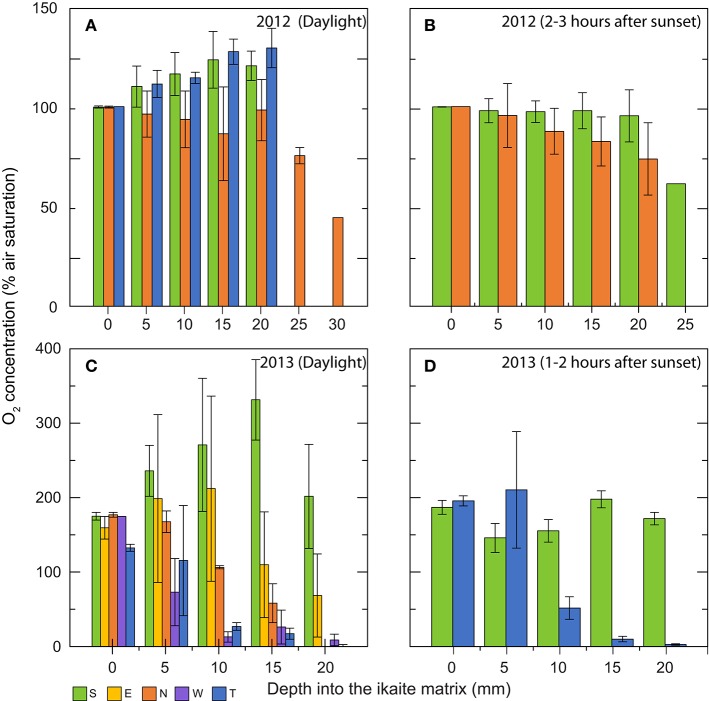
**Bar graphs showing *in situ* O_2_ concentration profiles (in % air saturation) measured at increasing depths into the ikaite matrix**. Data were obtained during two field campaigns: **(A,B)** in 2012 **(C,D)** in 2013. Graphs show daylight measurements **(A,C)**, and measurements performed 1–3 h after sunset **(B,D)**. Colors indicate measuring location on the ikaite tufa column; S = south, E = east, N = north, W = west, and T = top. Means ± SE (*n* = 3–25); *n* < 3 for bars without error bars. The wide span in the numbers of replicates is due to various factors (e.g., currents, cold-exposure, fatigue) making it difficult for the diver to remain steady during measurements in certain orientations for longer periods of time.

The O_2_ concentration profiles measured in the outermost 20–30 mm of the ikaite column apex displayed a great variability, not only from the column surface to deeper layers of the column, but also at different orientations on the column toward incident sunlight, as well as time of day (Figure 6). Although measurements were obtained at the same time of year, the ambient light conditions were rather different between 2012 and 2013. In 2012, dense fog often covered the fjord until midday, whereas foggy conditions were absent during the 2013 measuring campaign thus exposing the columns to more light prior to measurements. During daylight, a photon irradiance of ~100–300 μmol photons m^−2^ s^−1^ was measured at the column surface just before measurements in both years.

In 2012, O_2_ levels increased with depth in the ikaite matrix due to photosynthesis driving O_2_ concentration up to ~130% air saturation at the top and ~120% air saturation on the south side of the column apex at a depth of 20 mm inside the ikaite matrix (Figure 6A). In contrast, profiles of O_2_ concentration on the northern side of the column apex in 2012 showed a relatively constant decrease from 100% at the column surface to 44% air saturation at a depth of 30 mm inside the ikaite matrix, with measurements at 20 mm showing an O_2_ concentration close to 100% (Figure 6A). Measurements 1–3 h after sunset in 2012 (Figure 6B) showed a decrease in O_2_ on both the north and south side of the column apex, going from 100% at surface level to 60% air saturation at a depth of 25 mm on the south side, with a dramatic drop between 20 and 25 mm inside the column.

The O_2_ concentration profiles measured during the day in 2013 at the top of the ikaite column, facing directly toward the water surface, showed a slightly lower initial O_2_ concentration of 133% air saturation at the ikaite surface and a relatively fast decrease in concentration, going from 115% at 5 mm depth to 27% at 10 mm, where after it decreased constantly toward 0.4% air saturation at 20 mm depth below the ikaite surface. South-oriented measurements displayed an almost linear increase in O_2_ concentration from 175% at the ikaite surface to 330% air saturation at a depth of 15 mm below the surface before decreasing again to 202% at 20 mm depth. Measurements performed on the east-facing part of the ikaite column showed a similar trend albeit with slight lower O_2_ levels, going from an average of 160% saturation at the surface to 202% at a depth of 10 mm, followed by a stronger decrease to 69% air saturation at a depth of 20 mm. From measurements on the northern side during the day in 2013, we observed an almost linear decrease in O_2_ concentration with depth and the ikaite matrix exhibited anoxic conditions at 20 mm. On the west facing part of the column apex, we measured an even steeper decrease in O_2_ concentration, from 175% air saturation on the column surface to 13% at a depth of 10 mm (Figure 6C). From about 10–20 mm inside the column surface, we measured low O_2_ concentrations of < 27% air saturation. Night values in 2013 showed a relatively stable O_2_ environment with depth inside the column around a level of 150–200% air saturation, whereas measurements performed on the top decreased to almost anoxic conditions 15–20 mm inside the column. O_2_ levels of ~100% air saturation were measured in the surrounding seawater (data not shown).

**Figure 7:**
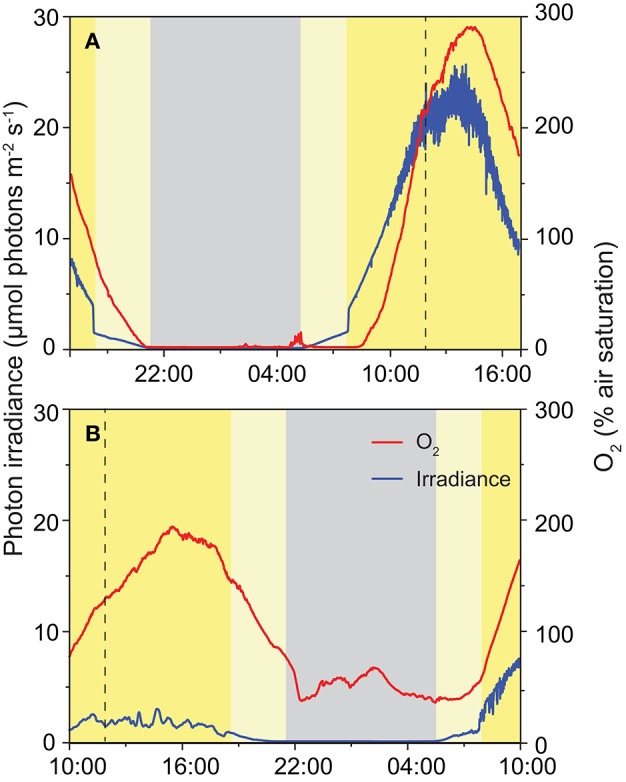
**Diurnal variation in photon irradiance (blue), and O_2_ concentration (red) measured during the field campaign in 2013 *in situ* ~15 mm inside the ikaite matrix at the apex of an ikaite tufa column at a depth of 7 m below the surface at mean sea level**. Panel **(A)** was obtained during clear skies, and **(B)** on a fully overcast day. Dashed vertical lines indicate noontime (12:00), gray area denotes night-time, light yellow area indicates indirect light just after sunrise and before sunset, dark yellow indicates time periods where the sun was clear from the mountains directly illuminating the fjord.

In 2013, changes in O_2_ concentration were recorded at a depth of 15 mm inside the ikaite matrix of the column apex with accompanying irradiance measurements on the ikaite surface during two distinct 24 h cycles; one with clear skies and one with 100% cloud cover (Figure 7). Under a clear sky and maximal sun exposure, high photosynthetic O_2_ production was observed driving O_2_ concentration in the ikaite to almost 300% air saturation and with photon irradiance levels (PAR, 400–700 nm) reaching 20–25 μmol photons m^−2^ s^−1^ at a depth of 15 mm inside the column during midday. With decreasing irradiance, we observed a steep drop in O_2_ concentration rapidly reaching ~0% air saturation just after sunset. In the morning, O_2_ levels in the ikaite matrix increased again with a short delay after sunrise (Figure 7A). The steep drop and rise in light levels seen around sunset and sunrise, corresponded to times where the sun disappeared behind the steep mountains in the west and appeared again in the east, respectively.

During an overcast day, photon irradiance in the ikaite matrix, did not exceed 3 μmol photons m^−2^ s^−1^ at a depth of 15 mm inside the column on the first day. However, we still observed a marked increase in O_2_ concentration during daytime reaching levels close to 200% air saturation. During this 24 h measuring cycle, we did not see the development of anoxia in the ikaite matrix during the night. After sunrise on day 2 (Figure 7B), there was less cloud formation and we observed the beginning of a similar trend in higher irradiance and O_2_ levels as observed in Figure 3A.

### *In-situ* measurements of photosynthesis

**Figure 8:**
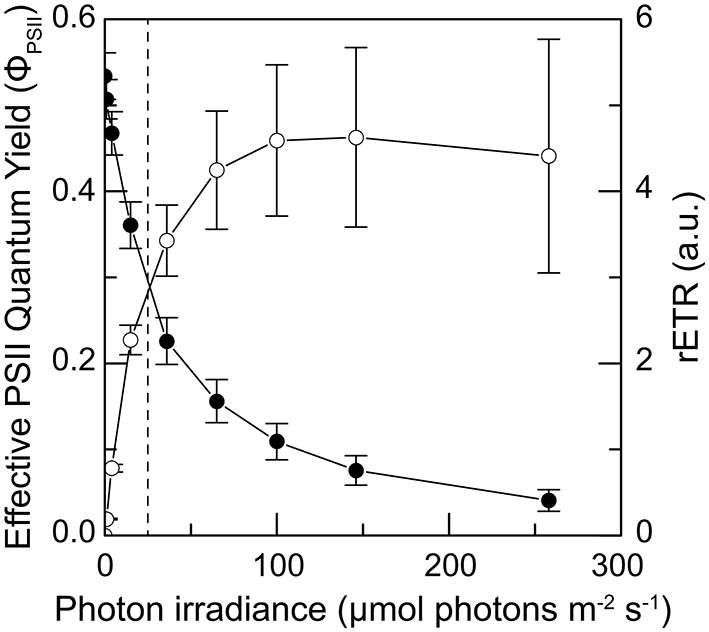
**Photosynthesis vs. irradiance curves obtained as 10 s rapid light curves of the effective quantum yield of PSII (solid symbols) and the relative electron transport rate (open symbols) measured as a function of increasing irradiance *in situ* ~15 mm inside the ikaite matrix at the apex of an ikaite tufa column at a depth of 7 m below the surface at mean sea level**. Dashed line indicates the maximal *in-situ* photon irradiance 15 mm inside the ikaite matrix. Symbols with error bars represent means ± SE (*n* = 11). See also Figures 3E,F.

Scraping away the outermost ~15 mm ikaite matrix to expose the underlying pigmented zones, gas bubbles, presumably originating from intense photosynthesis, were sometime released from the ikaite matrix. In August 2013, *in situ* variable chlorophyll fluorescence measurements showed an active photosynthetic community in the ikaite matrix that responded to increasing irradiance with a saturation response typical for microalgae and cyanobacteria (Figure 8). The effective PSII quantum yield exhibited high initial values indicating an active community of microbial phototrophs within the ikaite matrix. We observed a relatively low light saturation coefficient, E_k_, at a photon irradiance of ~24 μmol photons m^−2^ s^−1^, with rETR_max_ at 146 μmol photons m^−2^ s^−1^.

### Community composition

**Figure 9:**
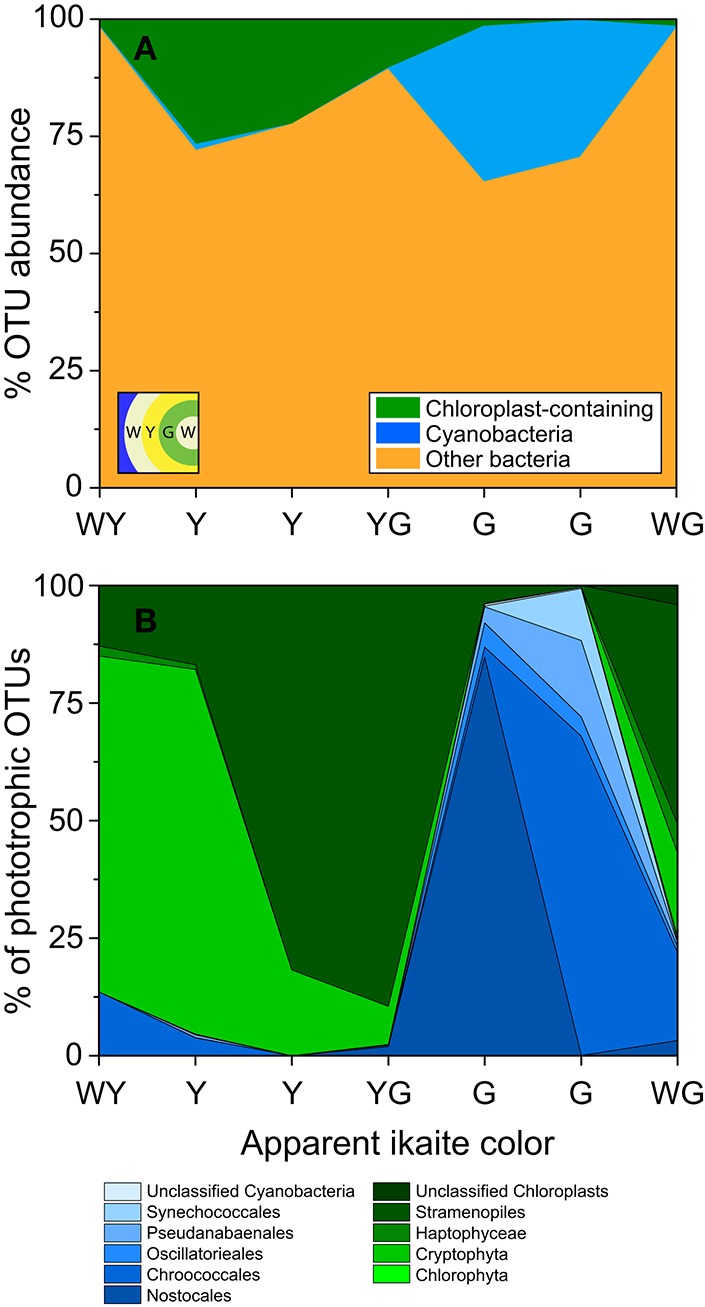
**Classification of microbial phototrophs in different colored layers within the ikaite matrix based on 16S rRNA gene pyrosequencing**. Samples were taken from different colored zones in the outermost layers of the ikaite column; white-yellow(WY), yellow (Y), yellow-green (YG), green (G), and white-green (WG). **(A)** Stacked area graph displaying the abundance of chloroplast and cyanobacterial OTUs in the seven colored zones. **(B)** Percent abundance of OTUs of oxygenic phototrophic origin, further divided between cyanobacteria and chloroplast containing organisms. Insert in **(A)** represent layering order in the ikaite.

The diversity of phototrophs in 7 ikaite samples was analyzed by pyrosequencing of 16S rRNA genes and revealed operational taxonomic units (OTUs) that could be assigned to cyanobacteria and microalgal plastids. At the phylum level, cyanobacterial OTUs represented about 0.2–33%, and microalgal plastids about 0.1–27% of all sequences from the ikaite samples, with microalgal plastids dominating in the outer yellowish ikaite zones and cyanobacteria dominating in the green zones found deeper in the ikaite, with only little overlap between the two zones (Figure 9A). Microscopic observation of fresh ikaite samples as well as macroscopic observations of ikaite cross-sections showed that ikaite crystals and cells in the pigmented zones were commonly embedded in exopolymers (Supplementary Video 1).

A more detailed analysis of the distribution of different phototrophs across differently colored zones of ikaite revealed distinct community changes in composition of oxygenic phototrophs (Figure 9B, Table 1). The deeper zone, 1–2 cm inside the ikaite matrix was comprised of a community of unicellular cyanobacteria belonging to the orders *Synechococcales* and *Chroococcales* and the filamentous orders *Nostocales, Oscillatoriales*, and *Pseudanabaenales*. In the microalgal zone 0–1 cm inside the ikaite, we identified plastids of microalgae belonging to the phylum *Chlorophyta*, and orders *Cryptophyta, Haptophyceae*, and *Stramenopiles*. The green layers were dominated by cyanobacteria, whereas the white-yellowish, yellow, yellow-green and white-green layers were dominated by plastid-bearing organisms. *Nostocales* predominated in one of the two sampled green zones, and *Chroococcales* in the other. *Cryptophyta* dominated in the white-yellowish layer, and *Stramenopiles* dominated in the two sampled yellow and yellow-green layers.

**Table 1:** **The percentage frequency of the most abundant identifiable cyanobacteria and chloroplast orders**.

**Phylum**	**Order**	**WY**	**Y**	**Y**	**YG**	**G**	**G**	**WG**
Cyanobacteria (total)		13.6	0.0	4.6	2.4	96.0	99.6	24.4
	Nostocales	0.0	0.0	0.0	2.0	84.7	0.1	3.3
	Chroococcales	13.6	0.0	3.8	0.3	2.1	67.9	18.7
	Oscillatoriales	0.0	0.0	0.0	0.2	5.2	4.1	1.2
	Pseudoanabaenales	0.0	0.0	0.6	0.0	3.4	16.3	0.8
	Synechococcales	0.0	0.0	0.2	0.0	0.1	11.1	0.4
Unclassified cyanobacteria		0.0	0.0	0.0	0.0	0.4	0.2	0.0
Chloroplast-containing organisms (total)		86.4	100	95.4	97.6	4.0	0.4	75.6
Chlorophyta		0.0	0.0	0.0	0.0	0.0	0.0	0.8
	Cryptophyta	71.4	18.3	77.5	8.1	0.2	0.0	17.9
	Haptophyceae	2.1	0.0	1.0	0.0	0.0	0.0	6.1
	Stramenopiles	12.9	81.7	16.9	89.5	3.8	0.4	46.7
Unclassified chloroplast-containing organisms		0.0	0.0	0.0	0.0	0.0	0.0	4.1

## Discussion

Previous *in situ* studies of ikaite tufa columns have mostly focused on their geological characteristics (e.g., Hansen et al., 2011) and documentation of their morphology and distribution in Ikka Fjord (Seaman and Buchardt, 2006), while microbiological investigations have exclusively been based on retrieved samples for subsequent enrichment and cultivation attempts, as well as molecular inventories of the microbial diversity in ikaite samples. The latter have revealed the presence of a diverse microbial community in the ikaite columns with a variety of putative aerobic and anaerobic metabolisms, and several heterotrophic isolates have been obtained (Schmidt et al., 2006; Vester et al., 2013, 2014; Glaring et al., 2015). However, no actual measures of microbial activity *in situ* have been undertaken, and the microenvironmental conditions in the ikaite tufa columns have remained unexplored besides a few measurements of internal pH and temperature in older parts of the tufa columns (Hansen et al., 2011). In this study, we thus present the first evidence of a photosynthetically active community of microalgae and cyanobacteria and show pronounced spatio-temporal heterogeneity of the chemical microenvironment in the ikaite crystal matrix of the tufa column apex that represents a new extreme habitat for Arctic phototrophs.

Cross sections of the apex of ikaite tufa columns revealed the presence of dense green and yellowish pigmented bands and patches in the outermost ~2–3 cm of the porous ikaite matrix that roughly followed the outside topography of the column (Figures 3A,D). At the apex of the columns, ikaite crystals continuously precipitates as larger, up to mm-sized ikaite crystals making the matrix rather porous and brittle as compared to the older and harder parts of the column surface, where calcite and mono-hydrocalcite become predominant from recrystallization and overgrowth by calcifying algae which stabilize the columns down toward the base (Buchardt et al., 2001; Hansen et al., 2011).

The surface temperature of the ikaite column exhibited shifting temperatures that correlated with tidal patterns (Figure 4). Hansen et al. (2011) showed that the internal temperature of tufa columns apparently followed fluctuations of the surrounding water, albeit with some dampening effects in the ikaite matrix. The outer ikaite layers thus experience a shift from a variable temperature at the tufa column surface toward a more stable pore water temperature deeper inside the ikaite matrix due to the constant percolation of cold spring water.

As the external temperature fluctuates with the tide, so will the salinity of the water surrounding the tufa columns when seawater is mixed with freshwater runoff and meteoric spring water from the columns. As shown by Hansen et al. (2011), these fluctuations are not only apparent on a diurnal scale but also occurring on a larger scale throughout the year. However, we found no effects on the photosynthetic activity due to tide-dependent changes in temperature and salinity of the external seawater, which we speculate is because of a “buffering” effect of the microenvironment in the ikaite matrix from the constant seepage of spring water.

The highly alkaline meteoric water flows through internal channels and exits by seeping through the porous ikaite matrix at the column apex (Hansen et al., 2011). We measured pH gradients going from that of seawater (~pH 8.2) toward pH 10.5–11 about 20 mm inside the matrix (Figure 5) regardless of light levels and orientation of the column measuring points relative to the sun, indicating that the pore water pH is mainly determined by the seepage of spring water and not by biological processes. The pH changed drastically from a mean value of pH 8.25 in seawater to pH 9.1 on the column surface, indicating that the phototrophic community in the ikaite matrix thrives under alkaline conditions.

The distinct patchy coloration of the subsurface ikaite layers (Figure 3) suggested the presence of photopigments, and the presence of active microbial phototrophs was confirmed by *in situ* photosynthesis measurements and molecular analysis (Figures 6, 8, 9). The patchy distribution of phototrophs probably reflects a heterogeneity in the light microenvironment within the ikaite, where the size of crystals and density of the ikaite matrix as well as the orientation toward the sun on a column may alter the internal light microclimate dramatically. We speculate that light attenuation increases as the ikaite column matrix becomes denser, moving down from the apex toward the base of the column and from the outermost ikaite layers toward the column center. Strong light scattering effects enhancing local PAR close to the ikaite surface may also affect the light microenvironment of phototrophs in line with observations in coral skeletons (Magnusson et al., 2007) and gypsum crusts (Oren et al., 1995), but detailed light measurements in ikaite are needed to further resolve the internal light microclimate of the tufa columns.

Despite low light levels due to reflection and attenuation of incident light in the ikaite matrix, we observed distinct irradiance-dependent O_2_ dynamics in the outermost 2–3 cm of the tufa column apex, ranging from anoxia to hyperoxic conditions reaching up to 300% air saturation under the highest irradiance levels (Figures 6, 7). In line with these observations, accumulated gas bubbles were sometimes released from within the ikaite matrix.

The O_2_ distribution in the ikaite matrix is affected by (i) respiration, photosynthesis and bubble formation in the EPS bound crystal matrix, where the phototrophs reside, and (ii) the slow perfusion and mixing of the spring water through the outer ikaite crystal matrix that may also slowly push gas bubbles toward the exterior. We did not quantify or map the distribution of gas bubbles, but we speculate that such bubble formation might be induced in the pigmented zones, where phototrophs and ikaite crystals were bound together in EPS (Supplementary Video 1), that in biofilms and microbial mats is known to impede O_2_ diffusion and thus lead to local supersaturation of O_2_ (e.g., Kühl et al., 1996). Our *in situ* data clearly show that bubble formation can buffer the O_2_ dynamics between day and night, and as the extent of O_2_ formation is dependent on solar irradiance, the O_2_ dynamics and buffering due to bubbles at night time will depend on the actual daylight scenario prior to measurements. As mentioned above, measurements in 2012 were generally done under more overcast/hazy conditions as compared to 2013, and we speculate that this caused more supersaturation and bubble formation during daytime that lead to rather high persistent O_2_ levels in the ikaite matrix at nighttime in 2013. Clearly, more precise measurements of the water movement as well as more precise quantification of the origin and dynamics of gas bubbles relative to the EPS containing zones with phototrophs in the ikaite matrix are needed to further resolve the O_2_ dynamics in the ikaite tufa columns.

Despite structural heterogeneity in structure and flow within the ikaite crystal matrix and local accumulation of bubbles, the first *in situ* measurements clearly demonstrated photosynthetic O_2_ production in the ikaite matrix, where O_2_ concentration varied strongly with irradiance and depth into the ikaite matrix. These findings were further supported by variable chlorophyll fluorescence measurements (Figure 8), showing the phototrophic community to be low light adapted while apparently tolerating irradiance levels far exceeding levels experienced inside the ikaite matrix.

Our measurements were performed during August, and are presumably representative of photosynthesis in the tufa columns under maximum light availability. How the community of phototrophic microbes and other microbes in the tufa columns survive under very low light levels or even endure extended periods in darkness during the winter months remains to be studied. However, *in situ* measurements showed potential for O_2_ depletion with depth into the ikaite matrix. The outermost 2–3 cm of the ikaite tufa column may thus become anoxic under prolonged low light or dark periods potentially enabling the presence of anaerobic microorganisms. We speculate that the structure of the columns with the internal flow channels surrounded by the porous ikaite matrix can facilitate localized draw of oxygenated seawater into the system. In the present study focus was on oxygenic photosynthesis and oxic respiration, but it is possible to use the underwater instrumentation with other microsensors (e.g., for N_2_O and H_2_S), and such measurements might reveal more details on the zonation and dynamics of anaerobic processes in the ikaite matrix.

The microbial community within the ikaite tufa columns has previously been shown to be relatively diverse (including anaerobic phylotypes) and with a clear difference in the overall taxonomic composition between the interior and surface parts of the column (Stougaard et al., 2002; Schmidt et al., 2006; Glaring et al., 2015). Glaring et al. (2015) showed that the surface and newly formed ikaite is dominated by cyanobacteria and phototrophic proteobacteria in contrast to the deeper layers dominated by heterotrophic bacteria. However, most studies were from deeper and older parts of the columns and our analysis thus provided a more detailed picture of the diversity and community composition in the outermost ikaite layers at the apex of the tufa columns, where differently pigmented zones were inhabited by distinct phototrophic communities and with only little overlap between zones dominated by cyanobacteria and microalgae, respectively (Figure 9A).

While the deeper green zone harbored a phototrophic community predominated by unicellular and filamentous cyanobacteria, the outer yellowish zones mainly harbored a diversity of microalgae (Figure 9B). Preliminary microscopic observations of fresh ikaite samples indicate that the yellowish zones in the ikaite columns are dominated by diatoms. Filamentous cyanobacteria, flagellated microalgae, and pennate diatoms are motile and would to some degree be able to move to optimal or less harmful conditions during dynamic changes in physico-chemical conditions in the ikaite, whereas unicellular cyanobacteria are immobile and more prone to have evolved other strategies to cope with fluctuating microenvironmental conditions. Along with the identified orders of cyanobacteria and microalgae, a large fraction of hitherto unidentified OTU's clustering with cyanobacteria was found in our molecular analysis. To further reveal the genetic and phenotypic characteristics of the microbial phototrophs, we have now initiated enrichment and cultivation attempts from fresh ikaite samples and a characterization of photosynthetic isolates is underway (Trampe et al., unpublished data).

Microscopic observation of fresh ikaite samples as well as macroscopic observations of ikaite cross-sections showed that ikaite crystals and cells in the pigmented zones were embedded in exopolymers (Supplementary Video 1) and several of the phototrophs identified in the molecular analysis of these zones are known to secrete mucilaginous organic compounds which may bind particles together (Kamennaya et al., 2012). The presence of EPS in the ikaite matrix seemed very extensive in the pigmented zones, however, more detailed studies on EPS in the ikaite columns are needed to get a better understanding of its possible role in the ikaite column strength and structural composition, along with potential O_2_ buffering properties mentioned above. As in photosynthetic biofilms (e.g., Kühl et al., 1996), the presence of exopolymeric substances (EPS) may slow down the solute exchange between the embedded active biomass and the surrounding pore water, which further exaggerates the formation of concentration gradients and chemical microenvironments in the EPS. The measured O_2_ gradients and dynamics in the ikaite matrix further support the notion that phototrophic communities in the ikaite tufa columns have characteristics of photosynthetic endolithic biofilms. We speculate that EPS secreted by the microbial biofilms within the ikaite columns may play an important role, affecting the microenvironmental conditions, not only in bubble formation and oxygen buffering by slowing down diffusion of O_2_ within the matrix, but also playing a role in column formation. We speculate that ikaite crystals are cemented together by biogenic EPS into a porous matrix strengthening and shaping the cementing processes involved in tufa column growth. It has been shown that ikaite crystals can easily be chemically precipitated in the laboratory (Bischoff et al., 1993). However, structures or larger aggregates of ikaite crystals cannot be induced by such simple chemical precipitation further pointing toward a hitherto unknown biological component, necessary in the column formation. Evidence for similar biological structuring has been found in lithified laminated microbialite structures formed by bio-stabilization via microbe-induced mineralization processes around EPS-trapped sediment particles (e.g., Basso et al., 2013). To resolve such mechanisms was beyond the scope of the present study, and the present finding that endolithic phototrophic biofilms prevail in the freshly-deposited ikaite crystal matrix should be followed up by more detailed studies of how these biofilms affect ikaite formation and/or deposition. More defined experimental systems using biofilm-forming cyanobacteria or diatoms cultivated from the tufa columns would allow better visualization, and quantification of crystal matrix formation relative to the EPS and microorganisms. Naturally, such defined biofilm systems would need to incorporate the physico-chemical gradients characteristic of the tufa column microenvironment.

## Conclusion

The uppermost parts of ikaite tufa columns in Ikka Fjord, Greenland, harbor active populations of microalgae and cyanobacteria embedded in exopolymers and stratified into yellowish and green zones, respectively, in the outermost ~2 cm of the porous ikaite matrix. *In situ* microenvironmental analysis revealed the presence of distinct O_2_ and pH gradients across the outermost 2 cm of the ikaite crystal matrix. pH increased from pH 8.2 in seawater to ~pH 9 at the tufa column surface and further increased to ~pH 10 1–2 cm below the surface, irrespective of ambient irradiance levels. Measurements of O_2_ concentrations in the ikaite showed pronounced dynamics with irradiance ranging from anoxia to hyperoxic conditions, albeit the porous ikaite matrix of crystals and phototrophs embedded in EPS sometimes exhibited accumulation of gas bubbles buffering such dynamics. Variable chlorophyll fluorescence measurements confirmed active photosynthesis in the pigmented layers of ikaite, which displayed low-light acclimation and high quantum yields *in situ*. The presence of dense pigmented layers of phototrophs and the observed chemical dynamics thus indicate the presence of endolithic biofilms inside the ikaite crystal matrix that may affect ikaite deposition and other biogeochemical processes in the tufa columns.

## Author contributions

ET and MK designed the study. Field work was carried out by ET, MK, and JL. All analyses were completed by ET, MK, MG, and PS. All authors contributed to the discussion of the results. Manuscript was written by ET and MK with inputs from MG, PS, and JL.

### Conflict of interest statement

The authors declare that the research was conducted in the absence of any commercial or financial relationships that could be construed as a potential conflict of interest.

## Supplementary material

Supplementary Figure 1 **Underwater photosynthesis measurements performed by a diver with a handheld underwater pulse amplitude chlorophyll fluorescence meter**.

Supplementary Video 1 **Video showing ikaite crystals with pennate diatoms embedded in an elastic EPS like substance**.
