# Supplementary figures and images for: In situ Dynamics of O2, pH, Light, and Photosynthesis in Ikaite Tufa Columns (Ikka Fjord, Greenland)—A Unique Microbial Habitat
Source: Front Microbiol. 2016 May 19;7:722. doi: 10.3389/fmicb.2016.00722 (PMC4871860; doi:10.3389/fmicb.2016.00722)

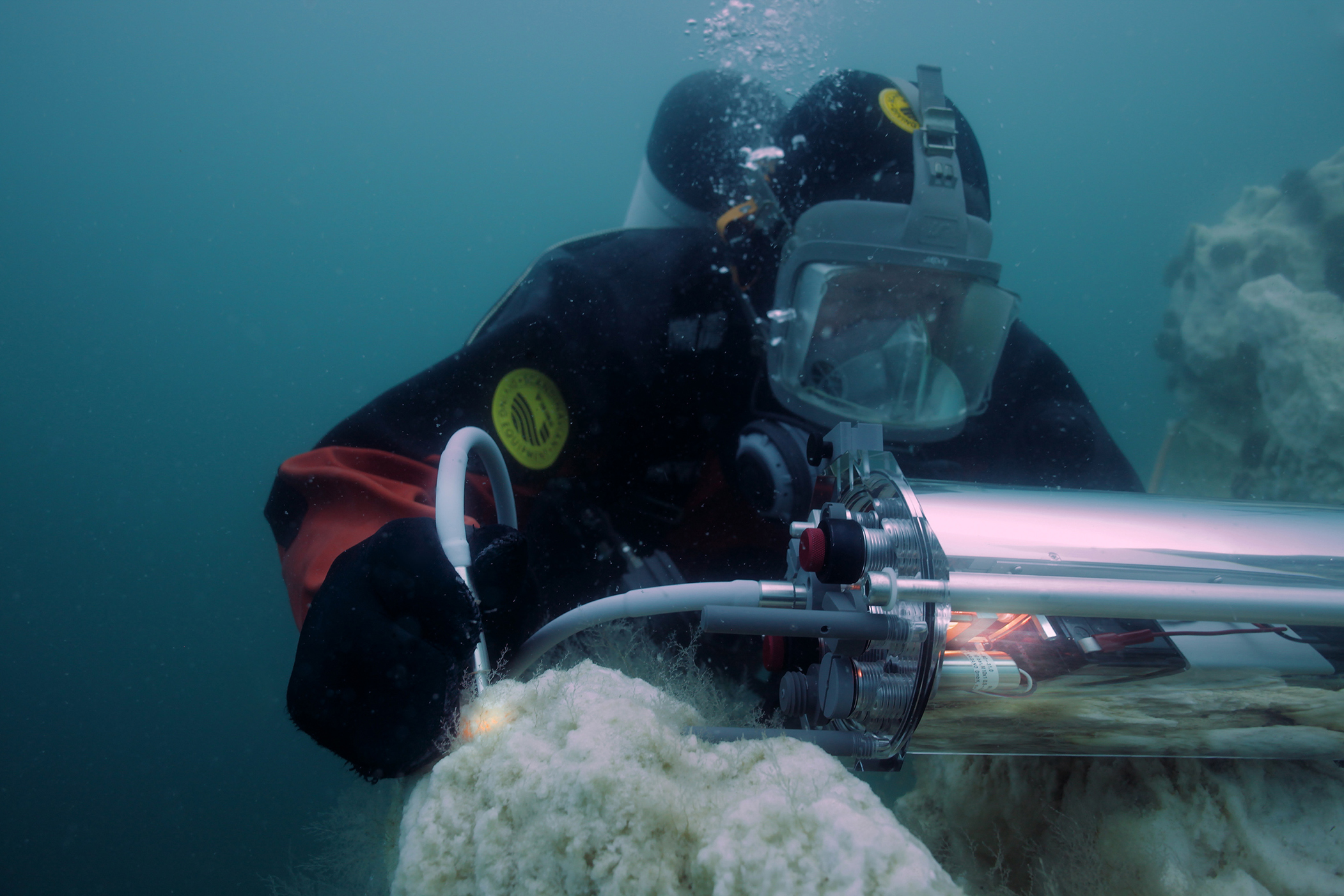

Supplement: Supplementary Figure 1 — Underwater photosynthesis measurements performed by a diver with a handheld underwater pulse amplitude chlorophyll fluorescence meter. [file Image3.JPEG]
